# Unusual Presentation of an Uncommon Disease: 24-Hour Cyclic Esotropia

**DOI:** 10.1155/2019/8602987

**Published:** 2019-03-05

**Authors:** Miguel Paciuc-Beja, Victor Hugo Galicia-Alfaro, Myriam Retchkiman-Bret

**Affiliations:** Centro Medico ABC, Mexico City, Mexico

## Abstract

Cyclic esotropia is an extremely uncommon form of strabismus. Classically, it follows a 48-hour cycle with 24 hours of orthotropia and 24 hours of manifest esotropia. We are reporting the first case of cyclic esotropia with a 24-hour cycle. A 7-year-old hyperopic female was seen with an esotropia of 6-month duration. Hyperopic glasses were prescribed by another ophthalmologist prior to our evaluation of the patient. While wearing her glasses, esotropia occurred during the morning and early afternoon. After 3.00pm, her eyes were “straight.” Many photographs were reviewed, confirming the esotropia pattern. Neurological evaluation was normal, including imaging and blood work to rule out thyroid pathology and myasthenia. After seeing the patient multiple times at different hours on different days, the diagnosis of cyclic esotropia was made. Bimedial recessions were performed with good results. The patient was still “straight” at distance and near 2 years after surgery.

## 1. Introduction

Cyclic esotropia was first described in the English literature by Burian in 1958 [1]. Duke-Elder [2] mentioned the “Cyclic Squint” as a “rare and curious condition which invariably takes the form of a temporary esotropia.” Most authors observed it as a 48-hour cycle [2–4]: 24 hours of orthotropia and 24 hours of esotropia. Costenbader reported a case that cycled every three to four days [5]. Cyclic esotropia is extremely rare: in 1964, Costenbader and Mousel calculated an incidence of one patient with cyclic esotropia in every 3000-5000 strabismic patients [5]. In 1966, Richter [6] reviewed thirty-three reported cases in the United States and England. Roper-Hall and Yapp [7] described two of the English cases in which changes in behavior and in EEG recordings were found during the “esotropic days.”

We are reporting a case of a 7-year-old girl with cyclic esotropia with a 24-hour cycle. She was esotropic from early morning until 2:00-3:00 pm and orthotropic the rest of the day. To the best of our knowledge, this is the first report of cyclic esotropia with a 24-hour cycle.

## 2. Case Report

A 7-year-old girl was seen with an esotropia of 6-month duration. According to the parents the esotropia was intermittent at first and became constant after several weeks. Another ophthalmologist prescribed glasses 4 months prior to our first appointment with the patient. With glasses on, she still had esotropia during the morning and early afternoon hours. Her eyes were “straight” after 3:00pm. No other signs or symptoms were present.

Our first examination was done around noon. She presented VA of 20/30 OU with her glasses of +5.50 sph OU. She measured with an esotropia of 50 PD at distance and 60PD at nearness, both with and without her glasses on. No diplopia was elicited. Anterior segment evaluation and dilated fundus exam were normal. Cycloplegic refraction with Cyclopentolate 2% showed +6.00 = +1.00 X 90 OU.

The mother had many photographs on her cell phone, which showed left esotropia in the morning (with glasses) and orthotropia (with glasses) in the afternoon, specifically after 3:00 pm.

Her next appointment was scheduled for late afternoon, at 5:00 pm. She was orthotropic at both distance and nearness with stereopsis of 100” of arc. Cycloplegic refraction with Atropine 1% showed the same refractive error as with Cyclopentolate 2%. This new prescription was dispensed.

Two weeks later, she was seen with the new glasses. She was esotropic at her morning appointment and orthotropic at her late afternoon appointment.

A neurological evaluation was performed which was normal. MRI was normal. Blood work to rule out thyroid disease and ocular myasthenia was also normal.

The diagnosis of cyclic esotropia was made. A 5.5 mm bimedial rectus recession was performed 6 months after her first appointment.

Postoperatively, she was orthotropic all day, with glasses. At 3-, 6-, 9-, and 12-month follow-ups, she was orthotropic. After 2 years, the patient is still orthotropic all day long.

Her prescription has changed to +4.00=+1.00X90 OU.

## 3. Discussion

The biologic clock refers to the regulation of physiology in cycles: sleep-wake cycle, core body temperature, hormone secretion, gene expression, proteins levels, enzymatic activity, and more.

Circadian mechanisms have been studied extensively. They play a significant role in “various aspects of metabolism and behavior,” with a more active role in lower animals than in humans [8].

Souza-Dias et al. [8] emphasize the scarcity of cyclic esotropia diagnosis by experienced strabismologists. The real incidence of cyclic esotropia is unknown. Not all cases are published. Perhaps some cases are undiagnosed due to rarity of the condition. In some published cases, while still cyclic in nature, the cases are secondary to ocular pathology or previous strabismus surgery.

In order to better understand cyclic esotropia, it would be helpful to separate the “primary” cyclic esotropias from other forms of cyclic strabismus. Do consecutive cyclic esotropia after surgery for intermittent exotropia and consecutive cyclic exotropia after surgery for adult-onset cyclic esotropia involve the same mechanisms as primary cyclic esotropia? To better understand the pathophysiology of this uncommon condition, it would be better to also set apart the “primary” cyclic esotropia cases from the cyclic esotropia cases secondary to previous ocular pathology or ocular surgery.

Cyclic esotropia differs from other circadian diseases in that peripheral manipulation of the extraocular muscles uniformly eliminates the problem. The cyclicity of the condition gives an excellent fusional potential. Richter postulates that ocular surgery “removes the hands from the clock without altering the clock itself” [6].

Primary cyclic esotropia occurrence is more frequent in children with hyperopia as in our case. The typical pattern is a 48-hour cycle. To the best of our knowledge this is the first case of cyclic esotropia with a 24-hour cycle.
